# An Adaptive Gate-Side Feedback Active Gate Driver for GaN Devices with Optimized Switching Performance

**DOI:** 10.3390/mi17070826

**Published:** 2026-07-10

**Authors:** Yuxin Zhang, Baoqiang Huang, Tiantian Wu, Zhe Wang, Qiao Zhang, Desheng Zhang, Jianming Lei, Run Min, Qiaoling Tong

**Affiliations:** 1 School of Integrated Circuits, Huazhong University of Science and Technology, Wuhan 430074, China; d202281038@hust.edu.cn (Y.Z.); d202387049@hust.edu.cn (B.H.); dszhangic@hust.edu.cn (D.Z.); leijianming@hust.edu.cn (J.L.); minrun@hust.edu.cn (R.M.); tongqiaoling@hust.edu.cn (Q.T.); 2 Beijing ZhiXin Microelectronics Technology Co., Ltd., Beijing 100000, China; wutiantian@sgchip.sgcc.com.cn (T.W.); wangzhe@sgchip.sgcc.com.cn (Z.W.); 3 School of Automation, Wuhan University of Technology, Wuhan 430074, China

**Keywords:** active gate driver, gate current control, GaN HEMT, slew-rate control, switching loss reduction

## Abstract

Gallium Nitride High Electron Mobility Transistors (GaN HEMT) are widely used in high-frequency applications owing to their fast-switching speed and low switching loss. However, the large dV_DS_/dt transients can cause severe crosstalk, current overshoot, and EMI issues. While conventional gate drivers can mitigate these issues by slowing down the switching process, the switching loss is significantly increased. To reduce the switching loss under dV_DS_/dt limitation, this paper proposes a gate-side feedback active gate driver (AGD) to adaptively regulate the switching transient by providing three-stage driving currents. Switching points of the three-stage driving currents are determined by detecting the start of the Miller plateau through gate voltage slope and identifying its termination when the gate voltage exceeds the Miller plateau voltage. With the detected signal, the gate driving current during the Miller plateau is reduced to suppress dV_DS_/dt and mitigate current overshoot. Under 400 V/20 A conditions, experimental results show that the proposed driver can effectively control dV_DS_/dt and suppress current overshoot, while reducing the dV_DS_/dt-related switching interval by 68.13% and switching loss by 51.8%, thereby confirming its effectiveness.

## 1. Introduction

With the rapid growth of modern power electronic systems, there is an increasing demand for converters with higher efficiency and power density. This trend has promoted the development of third-generation wide-bandgap (WBG) semiconductor devices. Among these technologies, GaN HEMT devices have attracted significant attention due to their superior material properties, including wide bandgap energy, high electron mobility, excellent thermal conductivity, and a high critical electric field. These characteristics allow GaN HEMTs to operate efficiently at high switching frequencies and high voltages. As a result, GaN-based devices have been widely adopted in many power conversion applications, such as high-frequency DC–DC converters [1], wireless power transfer systems, server power supplies, telecom power modules, and compact power adapters [2,3].

Unlike conventional silicon devices, GaN HEMTs rely on a two-dimensional electron gas (2DEG) [4,5,6] to form the conduction channel and therefore exhibit negligible reverse recovery loss. This characteristic enables GaN devices to operate at significantly higher switching frequencies. However, the fast-switching transitions also cause large current overshoot and increased EMI during the turn-on process. During switching transients [7,8], the rapid variation in V_DS_ and I_DS_, together with parasitic inductances and voltage-dependent device capacitances, can further aggravate these effects. Limiting the gate current during this interval can effectively reduce dV_DS_/dt and mitigate these issues. To achieve this, active gate driver (AGD) techniques provide multi-stage drive current during different switching phases, thus improving the switching performance of GaN HEMT.

Active gate driver approaches for GaN HEMT devices are typically divided into two categories: digital pre-set-type AGDs [9] and feedback-type AGDs [10,11,12]. Digital pre-set-type AGDs generally rely on open-loop circuit structures, which offer advantages in terms of simplicity and low implementation cost; however, they depend on prior characterization of device parameters and provide limited adaptability to varying operating conditions. Comparatively, feedback-type AGDs improve switching performance by actively shaping the device switching behavior through the adjustment of gate resistance, gate voltage, variable gate capacitance, or gate current [13,14,15,16]. These methods effectively control the charging and discharging processes of the device parasitic capacitances. Among the available approaches, gate current control enables finer regulation of the switching transient and is particularly suitable for integrated circuit implementations. Recent active gate-driver methods, such as [17,18], reduce switching overshoot by sensing dI_D_/dt or dV_DS_/dt and then adjusting the gate current or equivalent gate resistance. However, these methods mainly regulate the driving strength during detected transient intervals, while the accuracy of the gate-current switching instants is less emphasized.

Previous driving schemes are generally based on identifying the Miller plateau to enable driving current switching. In principle, the Miller plateau corresponds to the interval during which the gate voltage remains nearly constant while V_DS_ decreases. Due to parasitic capacitance coupling, the gate drive current directly influences the discharge rate of these capacitances, thereby affecting the slew rate of V_DS_. However, directly sensing dV_DS_/dt with high accuracy requires high-voltage capacitors and degrades the power density, making it necessary to detect alternative electrical signals to determine the onset of the Miller plateau. Moreover, due to the short duration of the Miller plateau in GaN devices, the detection and control delays severely degrade the performance of the AGDs. Under existing active gate driver techniques, precise switching of different gate-current levels cannot be guaranteed.

To overcome this limitation, an adaptive gate-side feedback strategy is proposed to identify the beginning and end of the Miller plateau across switching cycles. By utilizing the pulse-timing information extracted from the previous cycle to adjust the switching point in the current switching cycle, the proposed method relaxes the speed requirement of the detection circuit and gradually converges to the optimal current-switching instants cycle by cycle. As a result, accurate switching timings of the three-stage current are determined. Based on the switching timings, three-stage drive currents are applied during the turn-on transient to achieve dV_DS_/dt control. Experimental results further show that the proposed design achieves shorter switching times and lower switching losses compared with existing gate drivers.

The remainder of this paper is as follows. Section 2 analyzes the switching behavior of GaN devices and presents the proposed AGD architecture. Section 3 presents the circuit design details of the proposed AGD. Section 4 provides experimental results to verify the effectiveness of the proposed gate driver. Section 5 concludes the paper.

## 2. Switching Transient Analysis and the Proposed AGD

**Figure 1:**
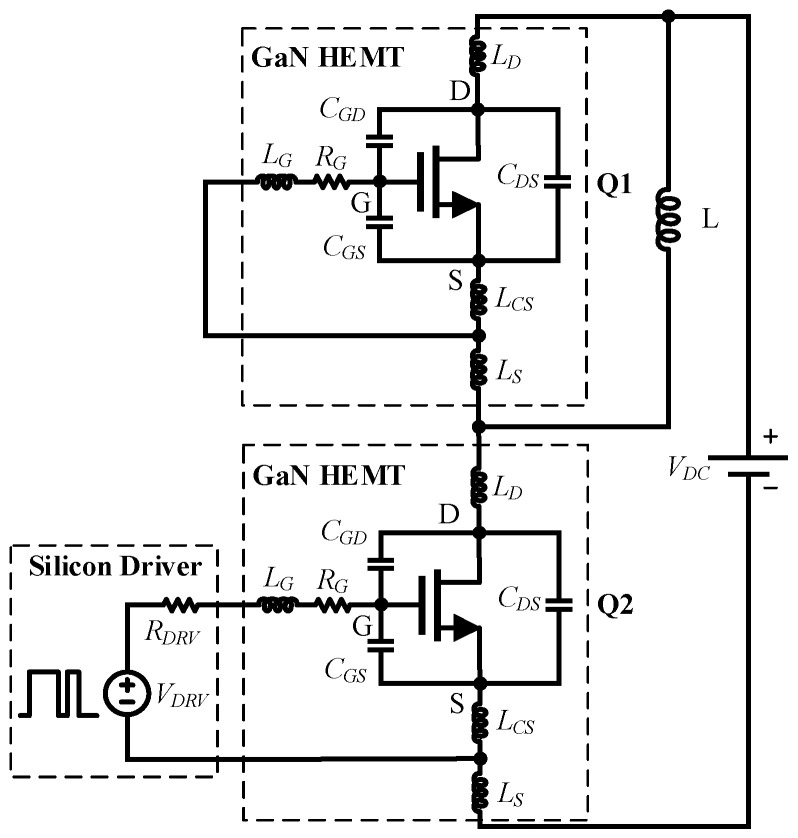
Double-pulse test circuit including parasitic parameters.

To establish the control logic of the proposed three-stage gate-driving scheme, it is first necessary to analyze the switching behavior of GaN HEMTs under double-pulse test (DPT) conditions. The DPT configuration, as shown in Figure 1, is widely adopted to characterize the dynamic switching performance of power devices by independently observing turn-on and turn-off transients under a controlled load current. In this setup, Q2 is the device under test, while Q1 operates as a freewheeling diode with its gate and source shorted.

### 2.1. Turn-On Transient Analysis

**Figure 2:**
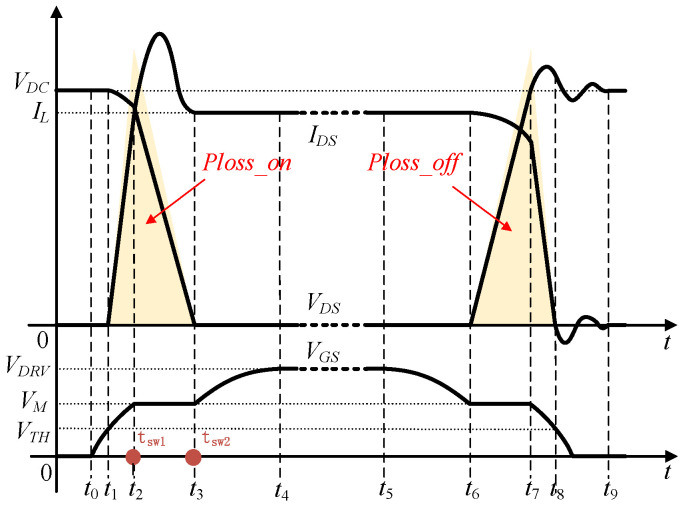
Waveforms of GaN HEMT switching process.

The turn-on transient of the GaN HEMT, illustrated in Figure 2, can be divided into four distinct intervals: (1) gate pre-charging, (2) current rising, (3) voltage falling (Miller plateau), and (4) gate overdrive charging.

Gate pre-charging interval (t_0_–t_1_): When the PWM signal transitions high, the gate driver injects current into the input capacitance C_iss_ ≈ C_GS_, causing V_GS_ to rise toward the threshold voltage V_TH_. During this stage, the conducting channel has not been formed, and the drain current I_D_ remains negligible.

Current rising interval (t_1_–t_2_): Once V_GS_ exceeds $V_{TH}$, the GaN HEMT 2DEG channel is established, and the drain current I_DS_ increases rapidly until it reaches the load current I_D_ = I_L_. Meanwhile, the gate continues charging C_iss_. V_GS_ increases but has not yet reached the Miller plateau. Although V_DS_ remains close to the DC bus voltage, a slight decrease can be observed due to the parasitic inductive voltage drop caused by the increasing dI_D_/dt.

Voltage-falling interval (Miller plateau) (t_2_–t_3_): During this interval, V_GS_ remains nearly constant at the Miller plateau. The gate current is primarily diverted into the gate–drain capacitance C_GD_, supporting the discharge of V_DS_. Notably, C_GD_ is strongly voltage-dependent, and its nonlinear behavior introduces additional complexity in dV_DS_/dt regulation:

(1) $$\left|\frac{dV_{DS}}{dt}\right|\approx \frac{I_{gate}}{C_{GD,eq}}$$

(2) $$I_{co}=C_{oss}\left|\frac{dV_{DS}}{dt}\right|$$

These expressions show that the gate current I_gate_ during the Miller plateau determines the V_DS_ falling slew rate. Here, C_oss_ is the equivalent output capacitance of the GaN HEMT, mainly including C_DS_ and C_GD_. A larger dV_DS_/dt produces a larger capacitive current through C_oss_, thereby increasing the turn-on current overshoot I_co_.

Gate charging interval (t_3_–t_4_): Once V_DS_ falls close to its on-state value, the gate current continues to charge the gate–source capacitance C_GS_, driving V_GS_ from the Miller plateau to the final gate voltage. A sufficiently large gate current in this stage helps shorten the overall switching transition.

The dominant switching loss occurs during the t_1_–t_3_ interval. A large gate current is applied in the first and fourth stages to minimize the overall transition time, whereas the gate current in the second and third stages must be carefully regulated, as these intervals govern both the switching energy and the dV_DS_/dt behavior. In particular, during the third stage, the rapid decrease in V_DS_, driven by the displacement current through C_GD_, results in a large dV_DS_/dt, which induces significant I_DS_ overshoot and oscillation due to parasitic inductances. By reducing the gate current in this interval, the V_DS_ slew rate can be effectively limited, thereby suppressing current overshoot and EMI.

### 2.2. Turn-Off Transient Analysis

The turn-off transient from t_5_–t_9_ is also divided into four intervals: (1) gate discharge, (2) voltage rising, (3) current falling, and (4) turn-off completion.

Gate discharge interval (t_5_–t_6_): When the PWM signal goes low, the gate driver removes charge from C_iss_, causing V_GS_ to decrease while I_DS_ remains approximately constant.

Voltage rising interval (t_6_–t_7_): Once V_GS_ reaches the Miller plateau V_M_, the drain current remains nearly constant, while V_DS_ increases rapidly toward the DC bus voltage.

Current falling interval (t_7_–t_8_): The drain current I_D_ decreases rapidly, and the resulting high di/dt may induce voltage overshoot due to parasitic inductances. Meanwhile, V_DS_ rises toward the DC bus voltage as the current decreases. Finally, as V_GS_ falls below the threshold voltage, the device enters the turn-off completion interval and becomes fully off.

Turn-off completion interval (t_8_–t_9_): V_DS_ remains at the bus voltage while V_GS_ decreases to the negative or zero gate bias. The device is fully turned off, and the switching transition is completed.

The dominant switching loss during turn-off occurs in the t_6_–t_7_ interval. Although limiting the gate current in the intermediate stages can suppress di/dt-induced voltage overshoot, this effect is less pronounced in GaN devices due to their inherently small parasitic inductance. In practice, the turn-off transition is primarily governed by the discharge of device capacitances rather than the gate driver. Therefore, the turn-off process is less sensitive to gate current shaping, and a strong pull-down capability is sufficient to ensure fast switching.

### 2.3. The Proposed Gate-Side Feedback Active Gate Driver

Based on the above analysis, the key challenge in three-stage gate driving lies in accurately determining the switching boundaries associated with the Miller plateau during turn-on. In conventional AGDs, these boundaries are detected in real time, which becomes increasingly inaccurate in GaN devices due to the extremely short duration of the Miller plateau.

**Figure 3:**
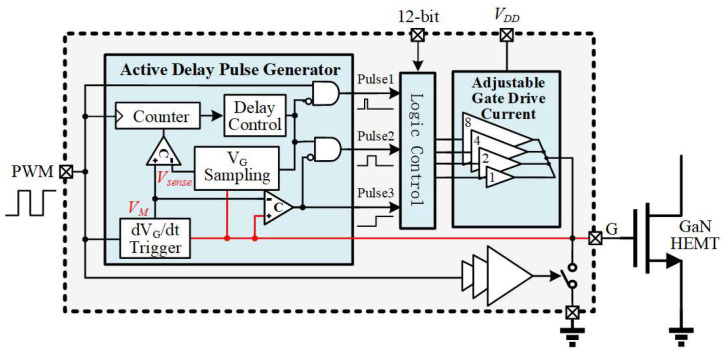
The structure diagram of the GaN HEMT AGD scheme.

To address this issue, this paper proposes an adaptive gate-side feedback active gate driver (GSF-AGD), as shown in Figure 3. The proposed gate driver provides three-stage driving currents during the turn-on transient to control the dV_DS_/dt by detecting the gate voltage and identifying the Miller plateau.

**Figure 4:**
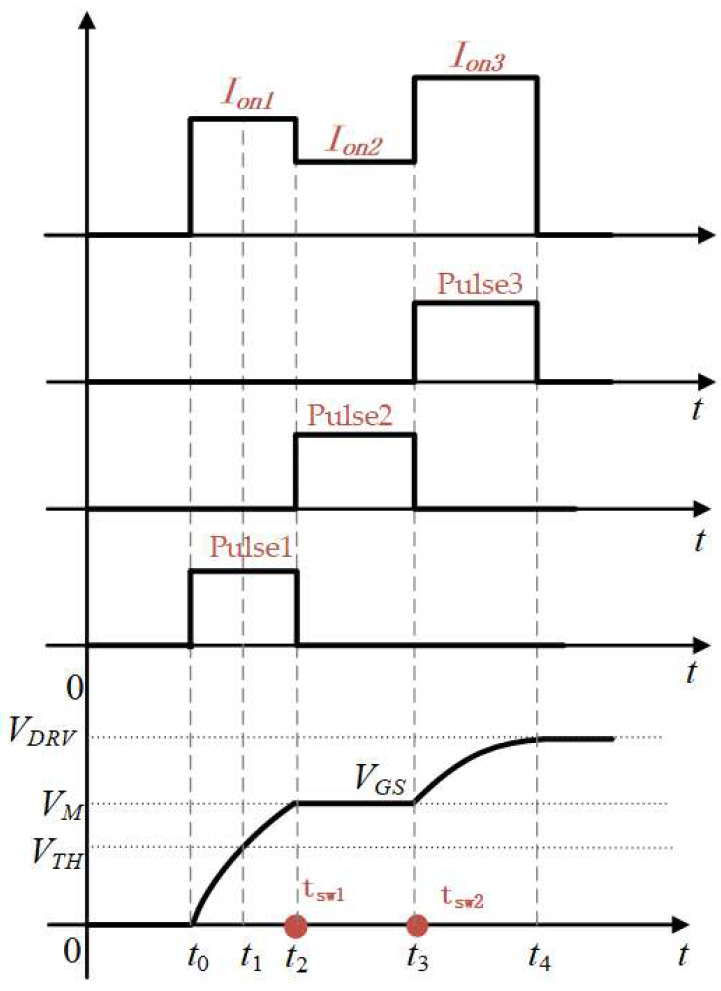
Gate-current and pulse timing diagram of the proposed design.

The operation principle of the proposed gate driver is shown in Figure 4. During *t*_0_–*t*_sw1_, a large driving current *I*_on1_ is provided to reduce the switching loss during the current rising stage. During *t*_sw1_–*t*_sw2_, a small driving current is applied to control dV_DS_/dt and suppress the current overshoot. During *t*_sw2_–*t*_4_, a large driving current is applied to reduce the switching delay. Due to the extremely short switching transient in GaN devices, real-time detection of the Miller plateau within a single cycle is inherently inaccurate. To address this limitation, a cross-cycle feedback adaptive strategy is adopted, in which the switching instants are predicted using information extracted from the previous cycle. Specifically, the onset of the Miller plateau is detected through a gate voltage slope sensing circuit, and the corresponding Miller voltage *V*_M_ is sampled and stored. In the subsequent cycle, this information is used to iteratively adjust the duration of Pulse1, enabling the switching instant to converge to the optimal boundary of the Miller plateau.

Once the plateau ends, identified by comparing the instantaneous gate voltage with the stored V_M_, the driver transitions to the final stage, where a larger gate current is applied to complete the turn-on process. As a result, a three-stage gate-current profile is achieved, where high current is applied before and after the Miller plateau to minimize switching time, while a reduced current is used during the plateau to regulate dV_DS_/dt and suppress current overshoot.

To realize the above adaptive timing control, the switching instant t_sw1_ must be accurately identified. Specifically, the rising edge of Pulse2 triggers the gate voltage sensing circuit to sample the gate voltage, generating V_sense_. The sampled voltage is then compared with the reference Miller plateau voltage V_M_. If V_sense_ < V_M_, it indicates that Pulse1 ends too early and the switching instant has not yet reached the Miller plateau; therefore, the up–down counter in Figure 3 increases by 1 LSB to extend the Pulse1 width in the next switching cycle. Conversely, if V_sense_ > V_M_, it indicates that Pulse1 ends too late and exceeds the optimal boundary of the Miller plateau onset; in this case, the counter decreases by 1 LSB to shorten the Pulse1 width in the next cycle. Through this multi-cycle adaptive update, the delay gradually converges such that Pulse1 turns off when the gate voltage reaches the Miller plateau voltage V_M_, thereby determining the optimal t_sw1_.

The adaptive update of Pulse1 can be expressed as:

(3) $$\left\{\begin{array}{cc} t_{d,\mathrm{pulse}1}\left(T\right)=t_{d,\mathrm{pulse}1}\left(T-1\right)+t_{d,1\mathrm{LSB}} & \mathrm{if}\;V_{\mathrm{sense}}<V_{M}\\ t_{d,\mathrm{pulse}1}\left(T\right)=t_{d,\mathrm{pulse}1}\left(T-1\right)-t_{d,1\mathrm{LSB}} & \mathrm{if}\;V_{\mathrm{sense}}>V_{M} \end{array}\right.$$

where t_d,pulse1_(T − 1) is the Pulse1 width in the previous switching cycle. When the operating condition varies slowly, the adaptive loop converges within several switching cycles.

After t_sw1_ is determined, Pulse2 is enabled to output the reduced gate current I_G2_ for dV_DS_/dt and current-overshoot suppression during the Miller plateau. During this interval, the instantaneous gate voltage V_G_ is continuously compared with the sampled Miller plateau voltage V_M_. Once *V*_*G*_ exceeds V_M_, the corresponding instant is defined as t_sw2_, indicating that the Miller plateau has ended. At this moment, Pulse2 switches from high to low, the output of I_G2_ is terminated, and Pulse3 is activated to enable I_on3_, which rapidly drives the GaN HEMT into full conduction.

The three gate-current levels are programmed by a 12-bit external control word, which is divided into three 4-bit groups corresponding to I_on1_, I_on2_, and I_on3_, respectively. Each group independently defines the current amplitude of its associated phase, enabling separate optimization of the current-rising interval, the Miller plateau interval, and the post-plateau interval.

In the logic control module, Pulse1, Pulse2, and Pulse3 serve as phase-selection signals to activate the corresponding current branches. Specifically, Pulse1 selects I_G1_, Pulse2 selects I_G2_, and Pulse3 selects I_G3_. Therefore, the external digital control determines the amplitudes of the gate currents, while the on-chip adaptive timing loop defines their transition instants. This separation between current-amplitude programming and adaptive timing control provides enhanced flexibility for optimizing dV_DS_/dt, switching loss, and EMI under varying operating conditions.

In this design, four MOSFETs with different sizes are used to generate binary-weighted output currents with weights of 1, 2, 4, and 8 units. Under the same gate-control voltage, their different W/L ratios produce approximately binary-weighted driving currents. By enabling different combinations of these MOSFETs using a 4-bit digital control word, the driver provides 15 discrete non-zero current levels. The minimum non-zero current is approximately 64 mA for the code “0001,” while the maximum current reaches approximately 960 mA for the code “1111.”

Since this work mainly focuses on turn-on transient optimization, no adaptive control is introduced in the turn-off path. Instead, a large-size Si NMOS transistor is employed to rapidly discharge the gate to ground, providing a peak pull-down current of approximately 1.9 A.

## 3. Circuit Implementations of the Proposed Gate Driver

To implement the proposed GSF-AGD, several key circuit blocks are designed to enable accurate Miller plateau detection and reliable timing logic control. As shown in Figure 3, the system consists of a gate voltage differentiator, a sample-and-hold (S/H) circuit, a high-speed comparator, an up–down counter, a programmable delay unit, and a logic control module.

The differentiator and S/H circuit extract the Miller plateau information, while the comparator and counter realize the cross-cycle adaptive timing update. Based on this information, the programmable delay unit and logic control module generate the phase-selection signals (Pulse1–Pulse3), which coordinate the three-stage gate-current transitions.

### 3.1. Voltage Sampling and Hold (S/H) Circuit

To accurately capture the Miller plateau voltage V_M_ under high-speed GaN HEMTs switching conditions, a high-bandwidth and low-error sampling circuit is required. However, several nonideal effects significantly degrade the sampling accuracy and limit the achievable speed, including bandwidth limitation, channel charge injection, and clock feedthrough.

The bandwidth limitation arises from the switch on-resistance R_on_ together with parasitic capacitances such as C_GD_, C_DB_, and the sampling capacitor C_S_, which form a low-pass network. This effect restricts the ability of the circuit to follow fast voltage transitions, thereby reducing the accuracy of the captured signal. Since these parasitic components are voltage-dependent and cannot be completely eliminated, the circuit must be carefully designed to minimize their impact and ensure sufficient tracking speed.

Another critical source of error is channel charge injection. When the sampling switch turns off, the channel charge is redistributed to the source and drain terminals, introducing an input-dependent voltage error on the sampling capacitor. In addition, clock feedthrough, caused by capacitive coupling between the gate and drain of the switching device, introduces a signal-independent DC offset at the output node. These effects become more pronounced under high-frequency operation and must be mitigated to maintain sampling accuracy.

To address these issues, a CMOS complementary switch structure is adopted. By properly matching the NMOS and PMOS devices, the injected charges with opposite polarities can partially cancel each other, thereby significantly reducing the charge injection error compared to a single-transistor implementation. Meanwhile, the influence of clock feedthrough is also alleviated due to the symmetrical structure of the complementary switch. In addition, the device sizing and circuit topology are optimized to reduce the effective resistance and parasitic capacitances, improving the overall bandwidth of the sampling path.

**Figure 5:**
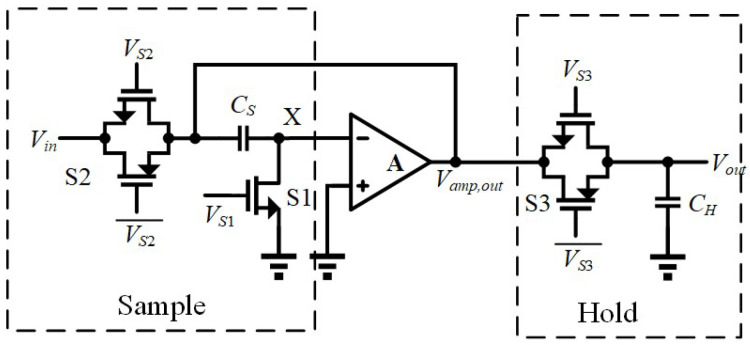
The proposed voltage sample-and-hold circuit.

The proposed sample-and-hold (S/H) circuit, shown in Figure 5, consists of a sampling network and a hold stage. During the sampling phase, switches S1 and S2 are turned on while S3 is turned off. One terminal of the sampling capacitor C_S_ is connected to the ground, and the other is connected to the input voltage V_in_. As a result, the voltage across C_S_ tracks the variation in the input signal, while the voltage across the hold capacitor C_H_, namely V_out_, retains the value from the previous sampling instant.

During the hold phase, switch S1 is first turned off, followed by S2, and then S3 is turned on. This switching sequence leaves the right plate of the sampling capacitor C_S_ floating, thereby preserving the charge stored at node X. Let V_0_ denote the input voltage at the instant when S1 is turned off. According to charge conservation, the amplifier output settles to the value determined by this stored charge, so both the amplifier output and V_out_ remain equal to V_0_ until the next sampling cycle. In this way, the Miller plateau voltage can be reliably captured and held for subsequent processing, enabling cross-cycle adaptive timing control.

**Figure 6:**
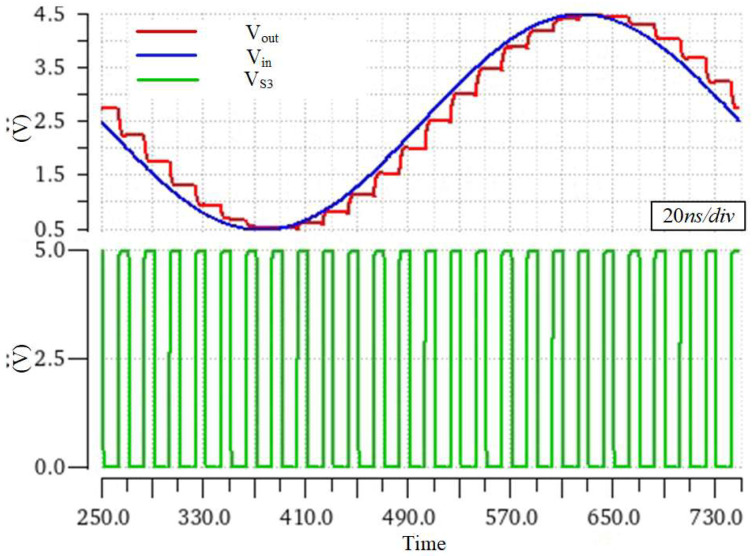
Simulated tracking performance of voltage sample-and-hold circuit.

The tracking performance of the proposed circuit is shown in Figure 6. In the simulation, the input signal of the S/H circuit has a frequency of 2 MHz, a DC level of 2.5 V, and a signal swing of 2 V. The sampling frequency is set to 50 MHz, corresponding to 25 sampling points in each signal period. The transient simulation results are presented in Figure 6. It can be seen that when the control signal of switch S3 in Figure 5 becomes active, that is, when S3 is turned on, the sampled output of the S/H circuit follows the input signal variation well. The circuit also exhibits a wide input and output voltage range. These results verify that the proposed S/H circuit can operate effectively under high-frequency switching conditions.

Overall, the proposed S/H circuit achieves high sampling accuracy, low offset, and fast settling behavior. The settling time is designed to be significantly shorter than the Miller plateau duration, ensuring reliable operation in high-speed GaN applications and enabling accurate extraction of switching information for the adaptive gate driver.

### 3.2. dV_GS_/dt-Triggered Detection Circuit

**Figure 7:**
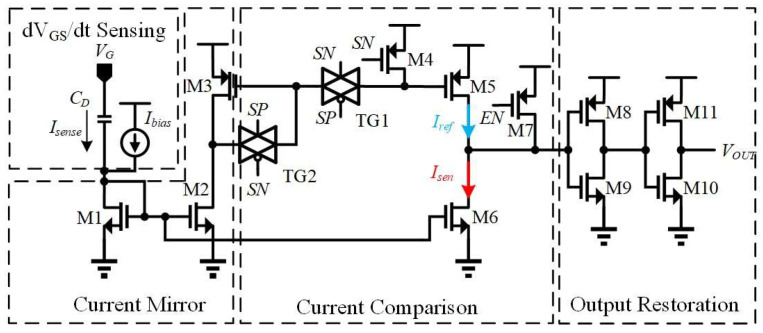
Schematic diagram of the slope detection circuit.

To enable accurate cross-cycle timing control, a dV_GS_/dt-triggered detection circuit is proposed to identify the onset of the Miller plateau. As shown in Figure 7, the circuit monitors the gate voltage V_G_ and detects the plateau by exploiting the fact that the slope dV_GS_/dt approaches zero during this interval.

Specifically, the gate voltage is first differentiated and converted into a current signal, which is then processed by a dynamic current comparator to generate a logic-level detection signal. When dV_GS_/dt decreases toward zero, the detection output transitions from high to low, triggering the sampling operation of the S/H circuit by controlling the timing of S1–S3. Once the slope increases again after the plateau, the detection signal returns high, and the circuit enters the hold phase. As a result, the captured voltage corresponds to the Miller plateau voltage V_M_, which is subsequently used for adaptive timing control.

**Figure 8:**
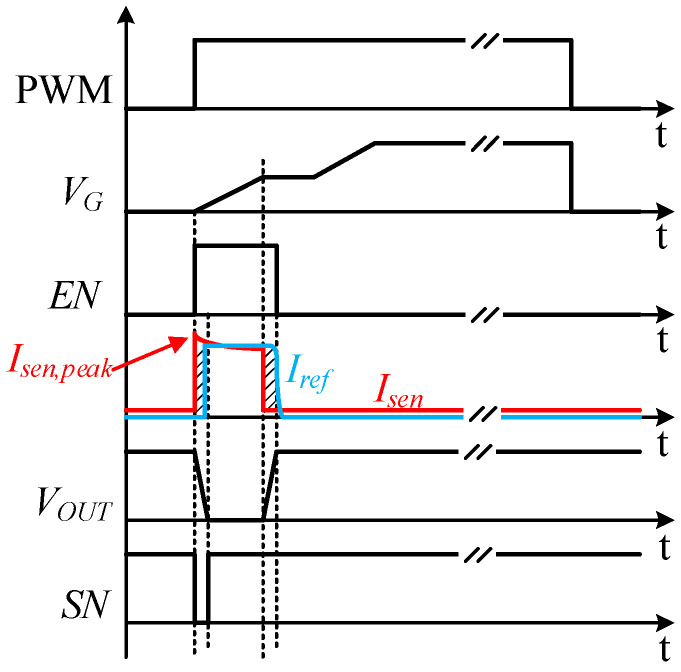
Timing diagram of the slope detection circuit.

The operation of the detection circuit is illustrated in Figure 8. After the PWM rising edge, a D-flip-flop chain and an XOR gate generate a narrow pulse, which is processed to produce EN and SN. The signal EN enables the detector for a short interval, preventing repeated triggering within the same PWM cycle.

At the beginning of detection, SN is low and SP is high. Thus, TG1 is off, while TG2 and M4 are on. TG2 diode-connects M3 to sample the sensing current, while M4 pulls the gate of M5 high and keeps the reference current branch off. Meanwhile, C_D_ converts dV_GS_/dt into I_sense_, which is mirrored to generate I_sen_. Subsequently, TG2 and M4 turn off, while TG1 turns on, transferring the sampled bias to M5 to generate I_ref_. M5 and M6 compare I_ref_ and I_sen_. When I_sen_ falls below I_ref_ near the Miller plateau, the comparison node changes state. The two cascaded inverters formed by M8–M9 and M11–M10 restore the logic level, sharpen the transition, isolate the comparison node, and provide sufficient drive capability for V_OUT_:

(4) $$I_{sense}=C_{D}\frac{dV_{G}}{dt}$$

**Figure 9:**
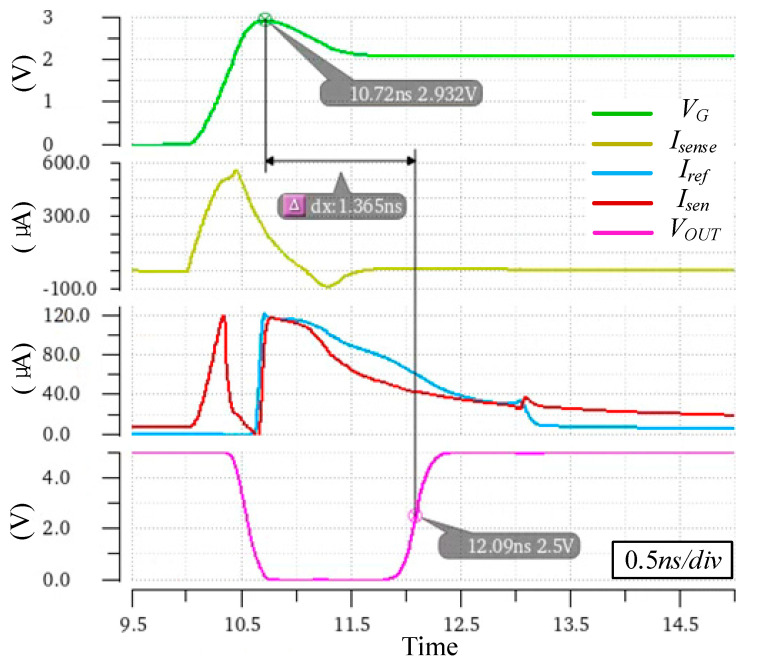
Simulation waveform of the slope detection circuit.

According to Figure 9, at 10.72 ns, the input voltage V_G_ of the slope detection circuit starts to rise rapidly. The differentiator converts the variation in V_G_ into Isense, which is further mirrored as I_sen_. At this moment, I_ref_ = 0; therefore, the comparison node is rapidly pulled down by M6, causing V_OUT_ to decrease quickly. As V_OUT_ decreases, M6 gradually leaves the saturation region, and I_sen_ decreases accordingly. When M6 enters the linear region, I_sen_ is further limited. Once the detection condition is satisfied, the control logic drives SN back to high and SP to low, turning off TG2 and turning on TG1. Meanwhile, M4 is turned off, and the gate voltage of M5 is held at the voltage corresponding to the detected peak current. As a result, I_ref_ quickly rises to approximately the maximum value of I_sen_. Since I_sen_ remains sufficiently large at this moment, M6 can still sink the reference current I_ref_, thereby keeping V_OUT_ low.

As V_G_ rises toward the Miller plateau, its slope gradually decreases, causing I_sense_ and the mirrored current I_sen_ to drop, while I_ref_ remains at the sampled peak value due to the S/H operation. Ideally, I_sen_ becomes much smaller than I_ref_, so the comparison node is pulled up and V_OUT_ recovers to 5 V. In simulation, I_sen_ does not fall completely to zero because of device nonidealities and changes in the operating region, resulting in a slight deviation from the ideal waveform. The two inverters before V_OUT_ are used as buffer stages to increase the driving capability and restore a full-swing digital output. The simulated response delay is about 1.365 ns, which does not affect the switching-point detection since V_DS_ has not yet entered its rapid falling interval.

When the Miller plateau is reached, the detection output changes state and enables the preceding S/H circuit to track the gate-voltage-related signal. After the plateau, as the gate voltage slope increases again, the detection output returns high, ending the sampling phase and preserving the captured value. Compared with direct dV_DS_/dt sensing, this method detects the switching point from the gate voltage behavior, avoiding high-voltage sensing devices and improving robustness under large dV_DS_/dt conditions.

### 3.3. High-Speed Comparator

**Figure 10:**
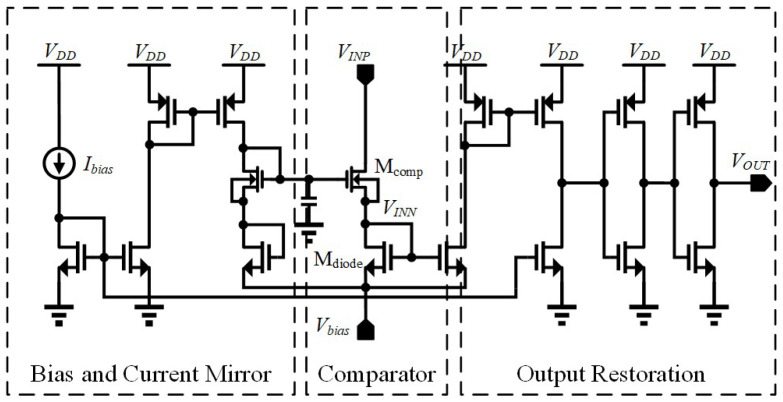
High-speed comparison circuit schematic diagram.

The proposed high-speed comparator, shown in Figure 10, is used to rapidly determine whether the input voltage V_INP_ is higher or lower than the reference voltage V_INN_. The circuit can be divided into three functional blocks: the bias/current-mirror block on the left, the core comparison block in the middle, and the output-buffer block on the right. The bias/current-mirror block generates and copies the required bias currents to ensure that the transistors operate under proper conditions. In the core comparison block, M_diode_ is diode-connected to establish the reference bias condition, while M_comp_ serves as the main comparison transistor. When V_INP_ < V_INN_, M_comp_ is weakly conductive or turned off, so the following internal node remains in its original logic state. When V_INP_ > V_INN_, M_comp_ turns on and produces a drain-current variation, which quickly changes the voltage of the internal node. As this current approaches saturation, M_comp_ behaves as a quasi-constant current source, allowing fast switching without excessive power consumption. The resulting voltage transition is then reshaped by the output-buffer stage into a clean digital output. This current-based comparison scheme avoids the large delay associated with multi-stage voltage amplification in conventional static comparators, making it suitable for high-speed GaN switching applications.

With deep negative feedback, M_comp_ and M_diode_ are sized proportionally to their bias currents, ensuring identical V_GS_. As a result, the source voltage of M_comp_ is stabilized at the reference voltage V_bias_, allowing accurate comparison between V_INP_ and V_bias_. In addition, the bulk of M_comp_ is tied to its source to eliminate body effect, improving comparison accuracy. The diode-connected transistor M_diode_ serves both as a current mirror and a reverse-current blocking element, preventing parasitic current paths that would otherwise increase power consumption.

Furthermore, the intrinsic gate capacitance of M_comp_ enhances circuit stability by suppressing disturbances coupled from rapid variations in V_INP_ through parasitic capacitances such as C_GD_.

**Figure 11:**
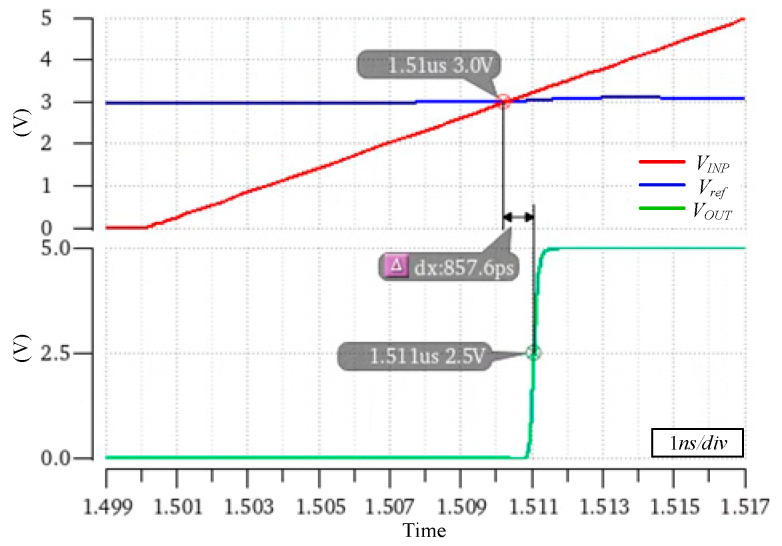
Comparator delay simulation results.

The delay performance of the proposed comparator is shown in Figure 11. With an input ramp from 0 V to 5 V within 17 ns and a reference voltage of 3 V, the comparator achieves a propagation delay of approximately 856 ps. This sub-nanosecond response confirms its suitability for high-speed GaN applications and enables accurate timing detection in the proposed adaptive gate driver.

## 4. Experimental Results and Analysis

To verify the effectiveness of the proposed driving strategy, the active gate driver was implemented using a 180 nm BCD process and evaluated under a 400 V/20 A operating condition. A double-pulse-test (DPT) platform was established using an enhancement-mode GS66508T GaN HEMT (GaN Systems, Ottawa, ON, Canada) as the power device, together with an evaluation board. Both system-level simulations and hardware experiments were carried out to validate the proposed design.

**Figure 12:**
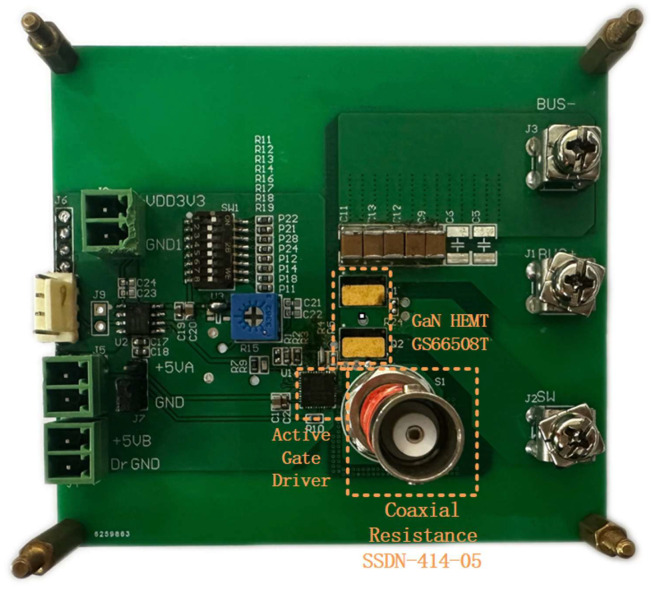
Double-pulse test board with an active gate driver.

A DPT prototype was built, as shown in Figure 12. To validate the effectiveness of the proposed optimized three-stage driving strategy, comparative experiments were performed on the MPT platform using a conventional gate driver (CGD), a conventional three-stage gate driver (CTSGD), and the proposed gate-side feedback active gate driver (GSF-AGD). All tests were conducted under an operating condition of 400 V/20 A. The proposed GSF-AGD was fabricated using a 180 nm BCD process, while a GS66508T GaN HEMT was employed in the power stage. A coaxial shunt resistor (SSDN-414-05) was used as the current-sensing element to provide a high-bandwidth current measurement port.

**Figure 13:**
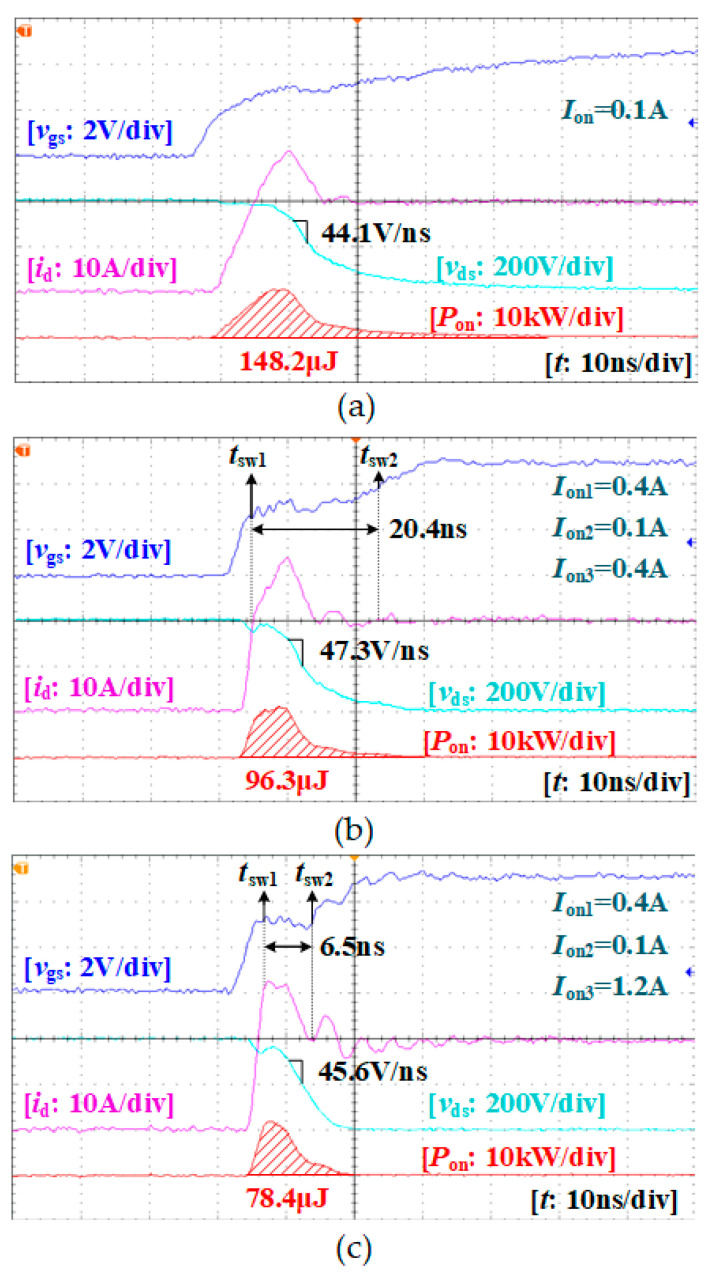
Comparison of switching waveforms of GaN HEMTs under 400 V/20 A conditions with reduced gate-driving strength: (**a**) CGD scheme; (**b**) CTSGD scheme; (**c**) GSF-AGD scheme.

Figure 13 compares the turn-on switching waveforms of the three schemes under 400 V/20 A conditions. Compared with the CGD and CTSGD, the proposed GSF-AGD achieves a significantly shorter voltage transition interval while maintaining the same current overshoot level. This indicates that the proposed design can simultaneously reduce switching loss while maintaining the same current overshoot. The improvement mainly comes from the adaptive phase-segmented gate-current control, which adjusts the gate current according to different switching intervals and optimizes the switching trajectory.

In the following experimental waveforms, the instantaneous turn-on power P_on_ is calculated from the measured drain-source voltage and drain current as:

(5) $$P_{on}\left(t\right)=v_{DS}\left(t\right)i_{D}\left(t\right)$$

The turn-on switching loss E_on_ is then obtained by integrating P_on_ over the turn-on interval, namely:

(6) $$E_{on}=\int P_{on}\left(t\right)d=\int v_{DS}\left(t\right)i_{D}\left(t\right)dt)$$

**Figure 14:**
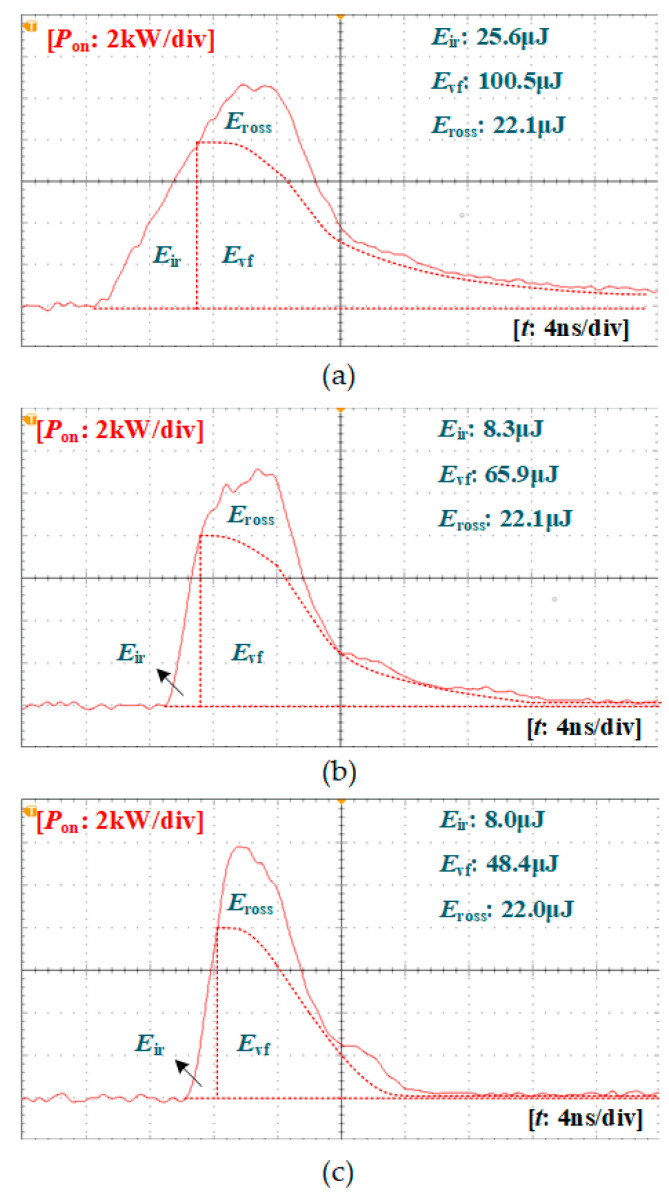
Comparison of switching loss of GaN HEMTs under 400 V/20 A conditions: (**a**) CGD scheme; (**b**) CTSGD scheme; (**c**) GSF-AGD scheme.

In this work, E_on_ is further divided into three parts: the current-rising loss E_ir_, the voltage-falling loss E_vf_, and the output-capacitance-related loss E_ross_. Therefore, the shaded area under the P_on_ waveform represents the total extracted turn-on loss, while Figure 14 further illustrates the contribution of each loss component.

For a conventional gate driver, reducing the gate-driving current is an effective way to suppress current overshoot and reduce dV_DS_/dt because the gate is charged more slowly and the drain-source voltage transition becomes gentler. However, this also extends the turn-on transition time and increases the overlap between V_DS_ and I_D_, resulting in larger turn-on switching loss. As shown in Figure 13a, although the conventional gate driver with a reduced driving current achieves a relatively low dV_DS_/dt of 44.1 V/ns, the turn-on energy reaches 148.2 μJ.

From the measured results, the total duration of the turn-on voltage transition is reduced from 20.4 ns in the conventional three-stage scheme to 6.5 ns in the proposed design, corresponding to a 68.13% reduction. This confirms that the proposed adaptive control can determine more suitable gate-current switching instants and achieve faster switching with improved transient behavior.

Figure 14 illustrates the switching energy distribution among the three types of gate driver designs. The main difference is observed during the voltage-falling interval. In the CGD scheme, the energy consumption in this interval is 100.5 μJ, which is reduced to 65.9 μJ in the CTSGD scheme, corresponding to a 34.4% reduction. Furthermore, in the proposed GSF-AGD design, the energy consumption is further reduced to 48.4 μJ, achieving a 51.8% reduction compared with CGD by optimizing the dV_DS_/dt transition period. Compared with the CGD scheme, both the CTSGD and the proposed GSF-AGD approaches exhibit a significant reduction in switching energy loss, with the proposed design achieving the most pronounced improvement.

**Figure 15:**
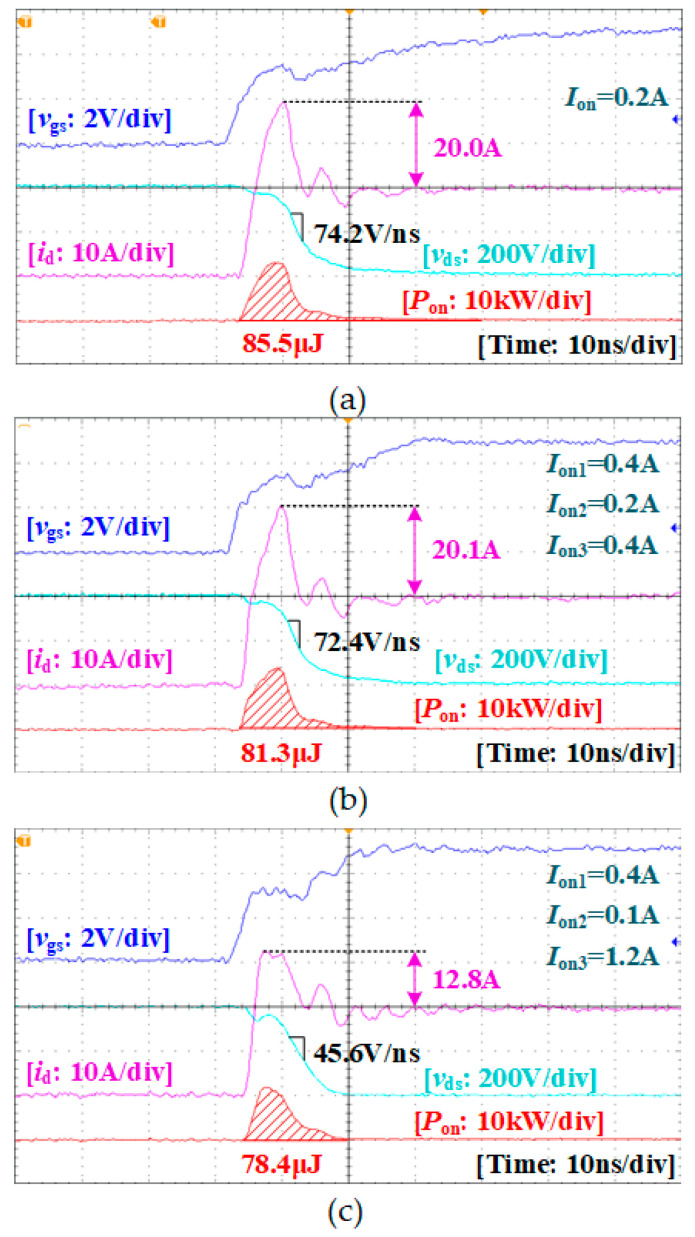
Comparison of switching waveforms of GaN HEMTs under 400 V/20 A conditions with increased gate-driving strength: (**a**) CGD scheme; (**b**) CTSGD scheme; (**c**) GSF-AGD scheme.

Furthermore, Figure 15 compares the switching waveforms of the GaN HEMT under different gate-driving strategies at 400 V/20 A. For a conventional gate driver, increasing the turn-on driving current is a common way to shorten the switching time and reduce turn-on loss because the gate is charged faster and the overlap duration between V_DS_ and I_D_ is reduced. However, this stronger driving capability also causes a faster V_DS_ transition, leading to a larger dV_DS_/dt and more severe current overshoot. As shown in Figure 15a, when the conventional gate driver uses I_on_ = 0.2 A, the turn-on loss is reduced to 85.5 μJ, but the dV_DS_/dt reaches 74.2 V/ns, and the current overshoot is about 20.0 A. Similarly, the conventional three-stage gate driver in Figure 15b achieves a switching loss of 81.3 μJ, but still exhibits a high dV_DS_/dt of 72.4 V/ns and a current overshoot of 20.1 A.

In contrast, the proposed GSF-AGD adaptively adjusts the gate-driving current according to the actual switching process instead of simply increasing the driving strength throughout the whole turn-on interval. As shown in Figure 15c, the proposed driver uses a smaller current during the Miller-plateau-related interval to control the V_DS_ falling slope, while applying larger currents before and after this interval to maintain fast gate charging. As a result, it reduces dV_DS_/dt to 45.6 V/ns and decreases the current overshoot to 12.8 A, while achieving the lowest turn-on loss of 78.4 μJ. These results demonstrate that the proposed adaptive control can break the conventional tradeoff between switching loss and switching stress, achieving lower loss, reduced current overshoot, and improved switching stability at the same time.

Analyses of Figure 13, Figure 14 and Figure 15 and Table 1 show that the proposed three-stage active gate driver can significantly reduce switching loss and shorten the switching interval. Under the same current overshoot condition, the proposed method achieves lower switching loss. Conversely, for a given switching loss, it results in reduced dV_DS_/dt and smaller current overshoot. These results verify the effectiveness of the proposed strategy in enhancing overall switching performance.

## 5. Conclusions

In conclusion, this work presents a gate-side feedback-based three-stage active gate driver for enhancement-mode GaN HEMTs. By tracking the Miller plateau voltage using gate voltage sensing and adaptive delay adjustment, the proposed driver accurately determines the switching boundaries. Accordingly, three-phase gate-current modulation is implemented, where higher currents are applied before and after the Miller plateau, and a reduced current is used during the plateau to regulate the switching trajectory. This approach enables effective dV_DS_/dt control and improves overall switching performance.

To validate the proposed scheme, the AGD chip was designed and implemented, and a double-pulse test platform was established for evaluation. Verification results demonstrate that the proposed driver can accurately identify the phase-transition instants and realize adaptive phase-segmented gate-current control. As a result, the proposed design effectively optimizes dV_DS_/dt, suppresses current overshoot, and reduces switching loss, achieving improved switching performance compared with conventional gate-driver approaches.

## Figures and Tables

**Table 1:** Performance comparison of three gate-driving approaches.

Gate Driving Approach	Strategy	dV_DS_/dt (V/ns)	E_on_ (μJ)
Conventional gate driver (CGD)	I_on_ = 0.1 A	44.1	148.2
	I_on_ = 0.2 A	74.2	85.5
Conventional three-stage gate driver (CTSGD)	I_on1_ = 0.4 A, I_on2_ = 0.1 A, I_on3_ = 0.4 A	47.3	96.3
	I_on1_ = 0.4 A, I_on2_ = 0.2 A, I_on3_ = 0.4 A	72.4	81.3
Proposed GSF-AGD	I_on1_ = 0.4 A, I_on2_ = 0.1 A, I_on3_ = 1.2 A	45.6	78.4

## Data Availability

Data are contained within the article.
